# Food Income and the Evolution of Forager Mobility

**DOI:** 10.1038/s41598-019-42006-2

**Published:** 2019-04-01

**Authors:** Elizabeth Gallagher, Stephen Shennan, Mark G. Thomas

**Affiliations:** 1 Research Department of Genetics, Evolution & Environment, University College London, Darwin Building, Gower Street, London, WC1E 6BT UK; 2 CoMPLEX, University College London, Physics Building, Gower Place, London, WC1E 6BT UK; 3 Institute of Archaeology, University College London, 31-34 Gordon Square, London, WC1H 0PY UK; 4 UCL Genetics Institute, University College London, Gower Street, London, WC1E 6BT UK

## Abstract

Forager mobility tends to be high, although ethnographic studies indicate ecological factors such as resource abundance and reliability, population density and effective temperature influence the cost-to-benefit assessment of movement decisions. We investigate the evolution of mobility using an agent-based and spatially explicit cultural evolutionary model that considers the feedback between foragers and their environment. We introduce Outcomes Clustering, an approach to categorizing simulated system states arising from complex stochastic processes shaped by multiple interacting parameters. We find that decreased mobility evolves under conditions of high resource replenishment and low resource depletion, with a concomitant trend of increased population density and, counter-intuitively, decreased food incomes. Conversely, increased mobility co-occurs with lower population densities and higher food incomes. We replicate the well-known relationships between mobility, population density, and resource quality, while predicting reduced food income, and consequently the reduction in health status observed in early sedentary populations without the need to invoke factors such as reduced diet quality or increased pathogen loads.

## Introduction

One of the obvious reasons foragers are mobile is their search for resources^1^, or better ones^2^, particularly in ecologies where resources can quickly become depleted or are slow to recover. However, in some ecologies (for example along coastlines) resources are more predictable and less easily depleted, or recover more quickly, and under these conditions foragers typically have reduced mobility^3,4^. Indeed, it can be seen that high mobility correlates with lower population density, lower food availability, less dependence on fish or higher effective temperature^3^.

When population density is high, food density can be relatively low due to depletion, and thus the net return of moving to another low quality resource patch can be less than that of staying put^5^. Furthermore, movement to high population density adjacent patches may be hindered. Thus, the Ideal Free Distribution (IFD) model predicts that on moving into a new environment individuals will select the highest quality resource patches, but as these fill up and interference competition increases they will obtain equally good pay-offs by moving to patches of a lower quality^6,7^. Marginal value theorem^8^ predicts that under these circumstances foragers should stay longer at patches, whereas when population densities are low relative to resources^5^ foragers are predicted to move frequently amongst patches. Similarly, when there is a strain on resource availability – perhaps because of high population density – foragers consider more patches in their decision of where to move, so the distance between patches is smaller and thus mobility again decreases^9^. Kelly^3^ suggests that since the presence of sedentary groups in a region removes possible resource areas from mobile groups, this might create a domino effect of switching to sedentism. The presence of low mobility groups in a region may also lead to a split between two successful strategies, a sedentary one exploiting the good patches and a mobile one exploiting those areas where sedentism is unviable.

On the other hand, in addition to resource procurement, mobility may facilitate other fitness enhancements in low density populations, including access to unrelated mates, and the establishment of more extensive social networks^10^. The latter can buffer against resource availability fluctuations^11^, increase resource knowledge to allow subsistence flexibility^12,13^, and maintain or increase culturally inherited technological complexity^13,14^. However, there are considerable costs to movement, including energy expenditure, planning, predation and other risks, opportunity loss, and time^4^ – all of which are likely to favour reduced mobility.

While local and neighbouring regional population densities have been implicated in mobility decisions, such densities must themselves be shaped by the same resource depletion and replenishment rates that influence mobility, but on a year-to-year rather than day-to-day time scale. The most dramatic change in mobility seen in the archaeological record is that associated with the transition from hunting-gathering to food production. This shift is associated with sedentism, an increase in population density^15^, and a reduction in health status^16^. However, the latter is widely seen not as a direct consequence of sedentism itself, but rather as a consequence of reduced dietary breadth and nutrient balance, and increased pathogen loads through increased population density, urbanization, proximity to animals, poor sanitation and long-distance trade^17–23^. While the innovations leading to full dependence on domesticated plants and animals are likely to be complex – including property rights and storage^24–26^ – any shift towards food production will slow resource depletion and increase replenishment rates.

Models can help to sharpen intuitions, reveal unexpected behaviours and test hypotheses, and are especially valuable when trying to understand past events where real time experiments are not possible, as is often the case in archaeology. In this study we develop a spatially-explicit agent-based cultural evolutionary simulation model to examine the extent to which mobility, population density and food income/health (as measured by non-cumulative food resource procurement in a single time step) are shaped by resource depletion and replenishment rates. Our model considers the feedback between people and their environment (as in^27^), and includes a culturally inherited and mutable mobility strategy, population growth, food income changes, and resource depletion and replenishment processes. It also includes movement decisions in an environment where resource availability varies (as in^2,27,28^).

## Model Overview

**Figure 1:**
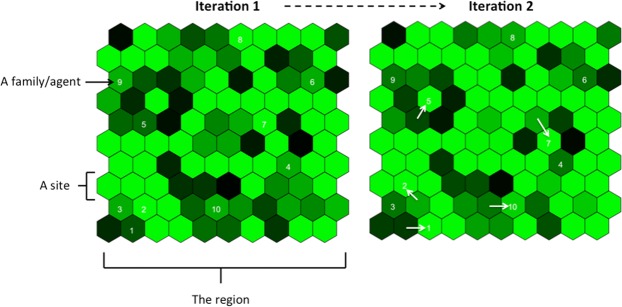
A visualisation of the model. Agents (white numbers) move from site to site (green hexagonals). The shade of the hexagonal reflects the foraging quality of the site – where the best quality is shown in bright green, and worst quality in dark green. The foraging qualities change from year to year according to natural growth and depletion.

**Figure 2:**
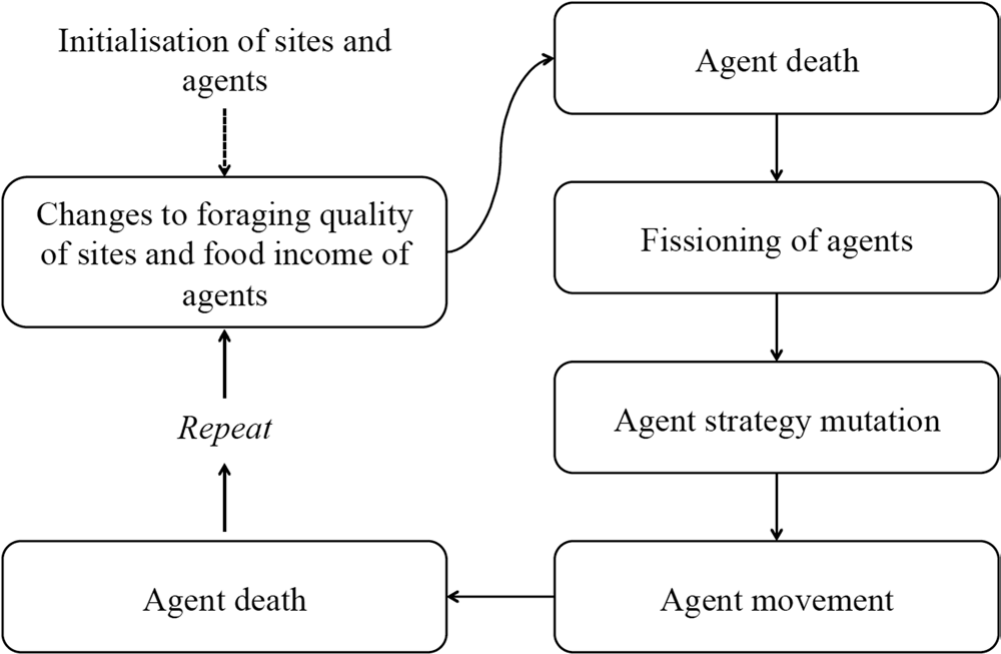
Stages of the model.

The Model simulates families moving around a region comprising a 2 dimensional grid of equally sized hexagonal sites. The model is iterative and each iteration is considered to represent one year. While we recognise that hunter-gatherer movements occur on an intra-annual basis, our model is intended to capture the overall mobility from year to year. Each site has a dynamic foraging quality, ${q}_{f}$ representing the food resources available. Family units (or ‘agents’) forage at one site in each iteration (multiple agents can co-occupy a single site). The agent’s foraging creates a feedback between the foraging quality of the site and the agent’s food income. The food income of an agent refers to short-term food procurement, and consequently well-being. It also affects the likelihood that an agent will fission or die, and so is related to fitness and population growth. It also has no cumulative (i.e. storage) or hereditary (i.e. passed on in an intergenerational manner) component. In this sense, an agent’s food income at the end of one iteration is not added to its food income in the next iteration, but rather it is recalculated based on the new environmental conditions. Agents have a mobility strategy, *m*, which is the probability of moving site in an iteration. Thus sedentary agents are those with *m* close to 0, and highly mobile agents are those with *m* close to 1. Agents can die, fission, mutate mobility strategy, and move from site to site, according to different probabilities. A visualization of two iterations of the model can be seen in Fig. 1 and the stages of the model are shown in Fig. 2.

Foraging resources stay within each site, and so do not include migrating animals. We assume there are two forces that act on the dynamics of a site’s foraging quality (${q}_{f}$): the natural replenishment rate, *r*, and a depletion scalar for when agents forage at the site, *λ*. We assume foraging quality grows logistically to a carrying capacity of 1, and that the amount of depletion is population density-dependent (as in^27,29,30^). Thus at iteration *t*+1 the foraging quality at a site is the natural replenishment minus depletion due to foraging, i.e.:

$${q}_{f,t+1}={q}_{f,t}+r{q}_{f,t}(1-\frac{{q}_{f,t}}{1})-\lambda (1-\frac{1}{n+1}){q}_{f,t}$$

where *n* is the number of agents occupying the site.

The food income of an agent changes within its lifetime, and is calculated at iteration *t* as:

$$f=\,\frac{{q}_{f,t-1}}{n}$$

where ${q}_{f,t-1}$ is the foraging quality in the previous iteration of the site the agent occupies. If $f < {f}_{min}$ then the agent dies. Hence, there is no accumulation of food income as the agent’s food income in the last iteration does not affect its current food income.

A successful family (agent) should be interpreted as one that is able to support its members, and hence a high food income level means an agent has procured sufficient resources to split, generating a new agent. When fission occurs the new agent occupies the same site as the parent agent, but is then free to move in the next iteration. The new agent will have the same mobility strategy as its parent and a randomly chosen initial food income value (reflecting the non-heritability of food income).

We assume that if an agent has the minimum food income (${f}_{min}$) then there would be no chance of fissioning. Using information from both^3^ and^31^ (see *SI Appendix*, section 1 for details of this data) we set the relationship between an agent’s food income and its probability of fissioning, *p*, as

$$p=\,{p}_{max}-\frac{{p}_{max}}{\mathrm{log}({f}_{min})}\,\mathrm{log}(f)$$

where data from^3^ indicates ${p}_{max}=0.14$ and ${f}_{min}=\frac{1}{{n}_{max}}$. See *SI Appendix*, section 2 for the details of these assumptions.

Following fission, all agents can then mutate their mobility strategy with probability $\mu \in [0,1]$ each iteration. This mutation could be thought of as random experimentation in cultural learning. We assume that approximately every generation, taken as 25 years, an agent changes the way it moves, and thus $\mu =1/25=0.04$.

Change in mobility strategy is informed by both the agent’s food income and previous strategy. We use a Binomial distribution to pick the new mutated strategy value from, with parameters chosen such that the strategy value will not change much if the agent has a high food income level, and conversely if the agent has a low food income level then the change will be less conservative. Thus, the rate of change in a cultural strategy is pay-off dependent – fitting with the idea of necessity being “the mother of invention”^32^ (but see^33^). We describe the assumptions and the distribution for this in *SI Appendix*, section 3.

The model allows for the evolution of the mobility strategy by natural selection, such that if, for example, a low mobility results in high agent food income, then agents with a low *m* value will be more likely to survive and fission (and so pass on their low *m* value) compared to agents with a high *m* value. Furthermore, we introduce variation into the population via the mutation step, so new and possibly more advantageous strategy *m* values can evolve. However, mobility strategies could emerge in a population randomly, or be set by the starting conditions, so to distinguish between the effects of selection on the one hand, and random cultural drift and starting conditions on the other, we introduce a ‘null strategy’. All agents have a null strategy that is initialized, passed on, and mutated in exactly the same way as the mobility strategy, but has no effect on behaviour or food income, i.e. the null strategy is randomly assigned and its value is unlinked to any agent behaviours. Thus we can compare the changes in null strategy with the changes in mobility strategy to see if there are any real selection effects.

Reflecting a key assumption of the IFD, agents are able to distinguish, and are more likely to move to, more ‘attractive’ sites. Site attractiveness is a relative measure of how close a site is, scaled by how fit an agent could be if it moved there, and ranges between 0 and 1 (full details are given in *SI Appendix*, section 4). Moving sites costs the agent an amount of food income scaled by the distance it moves. If an agent moves from site *a* to site *b* its new food income level is

$$f={f}^{\text{'}}-\eta {d}_{a,b}$$

where *η* is the cost of movement parameter, ${d}_{a,b}\,$is the distance between sites and ${f}^{\text{'}}$ was the agent’s food income before moving. We would expect that the outer sites in the region would be less densely populated than the inner sites since movement to inner sites is more likely.

Using ethnographic data^3^ we find that in a 10×10 region a realistic value for the maximum number of agents at a site, ${n}_{max}$, is 6 and the area of a site is 10 km^2^ (for full explanation see *SI Appendix*, section 5). There are ${\rho }_{init}{n}_{max}{s}_{x}{s}_{y}$ agents in the initial iteration of the model, where ${\rho }_{init}$ is the initial population density, ${n}_{max}$ is the maximum number of agents which can be supported at one site, and ${s}_{x}{s}_{y}$ is the number of sites in a region (i.e. 10×10). These initial agents are all randomly assigned a site to occupy, their food income is randomly assigned in $[{f}_{min},1]$, and their mobility strategy is randomly assigned in [0.01, 0.99]. The foraging qualities of sites are also randomly assigned in $[{q}_{f,min},1]$.

The 4 main steps for every iteration of the model are: Foraging quality and food income updates -> Fissioning -> Mutation -> Movement. In the first and last steps, death of unfit agents can occur (Fig. 2). All the parameters, constants and variables in the Forager Model are given in Table 1.

**Table 1:** Parameters, constants, and variables in the model.

Type	Symbol	Description	Default Value	Range	Value used or range varied in simulations
Parameter	*r*	Foraging quality replenishment rate	—	[0, 1]	[0, 1]
Parameter	*λ*	Foraging quality depletion scalar	—	[0, 1]	[0, 1]
Parameter	*κ*_*m*_	Mobility strategy conservatism	—	—	[50, 150]
Parameter	*p*_*max*_	Maximum probability of fission	0.14	[0, 1]	0.14
Parameter	*η*	Food income cost of movement per site	—	[0, 1]	[0, 0.1]
Parameter	*n*_*max*_	Maximum number of agents which can be supported at a site	6	≥1	6
Parameter	*μ*	Probability of mutation	0.04	[0, 1]	0.04
Parameter	*p*_*init*_	Initial population density	0.1	[0, 1]	0.1
Constant	*s*_*x*_	Number of sites in the x axis	10	—	10
Constant	*s*_*y*_	Number of sites in the y axis	10	—	10
Constant	*q*_*f,min*_	Minimum foraging quality	0.1	—	0.1
Constant	*N*_*max*_	Maximum number of families possible in the region	600	*n*_*max*_ *s*_*x*_ *s*_*y*_	600
Constant	*N*_*init*_	Total number of agents initially	60	${\rho }_{init}{n}_{max}{s}_{x}{s}_{y}$	
Constant	*f*_*min*_	Death food income level	0.17	$1/{n}_{max}$	0.17
Variable	*q*_*f*_	Site foraging quality	—	$[{q}_{f,min},1]$	
Variable	*m*	Agent mobility strategy	—	$[0.01,0.99]$	
Variable	*f*	Agent food income	—	$[{f}_{min},1]$	
Variable	*p*	Probability of fission	—	$[0,{p}_{max}]$	
Variable	*d*_*a,b*_	Distance between sites *a* and *b*	—	$[0,{d}_{max}]$	
Variable	*f* ^*^	Potential food income at a site	—	(0, 1]	
Variable	*A*	Attractiveness of a site	—	[0, 1]	
Variable	*n*	Number of agents at a site	—	$[0,{n}_{\max }]$	

## Results and Discussion

Of the 100,000 simulations performed, 98,514 had ≥15 agents in the final iteration. The 1486 simulations with <15 agents had very low values for the replenishment rate, *r*, high depletion rates, *λ*, and high costs of movement, *η*. Under these conditions agents are unlikely to be sustained long-term, even at low population densities. Correlation coefficients between parameters and outcomes are given in Table 2.

**Table 2:** Pearson’s moment correlation coefficients for each pair of parameters and outcomes.

	*r*	*λ*	*κ*_*m*_	*η*	Initial mean mobility strategy	Initial mean null strategy	Final number of agents	Final mean mobility strategy	Final mean food income	Final mean null strategy
*λ*	0.01									
*κ*_*m*_	0.00	−0.01								
*η*	0.01	0.00	0.00							
Initial mean mobility strategy	0.00	0.00	0.00	0.00						
Initial mean null strategy	0.00	0.00	0.00	0.00	0.00					
Final number of agents	0.39	−0.64	0.01	−0.42	−0.01	0.00				
Final mean mobility strategy	−0.23	0.64	−0.03	−0.16	0.08	0.00	−0.63			
Final mean food income	−0.24	0.52	0.01	0.43	0.01	0.00	−0.80	0.47		
Final mean null strategy	−0.01	0.00	0.00	0.00	0.00	0.21	0.00	0.01	0.00	
Final mean foraging quality	0.37	−0.38	0.01	0.68	−0.01	0.00	0.26	−0.59	−0.02	0.00

The 98,514 simulations which had ≥15 agents in the 1000^th^ iteration of the model were used to calculate these. Shade signifies strength of the correlation where red is for positive correlations and blue is for negative.

**Figure 3:**
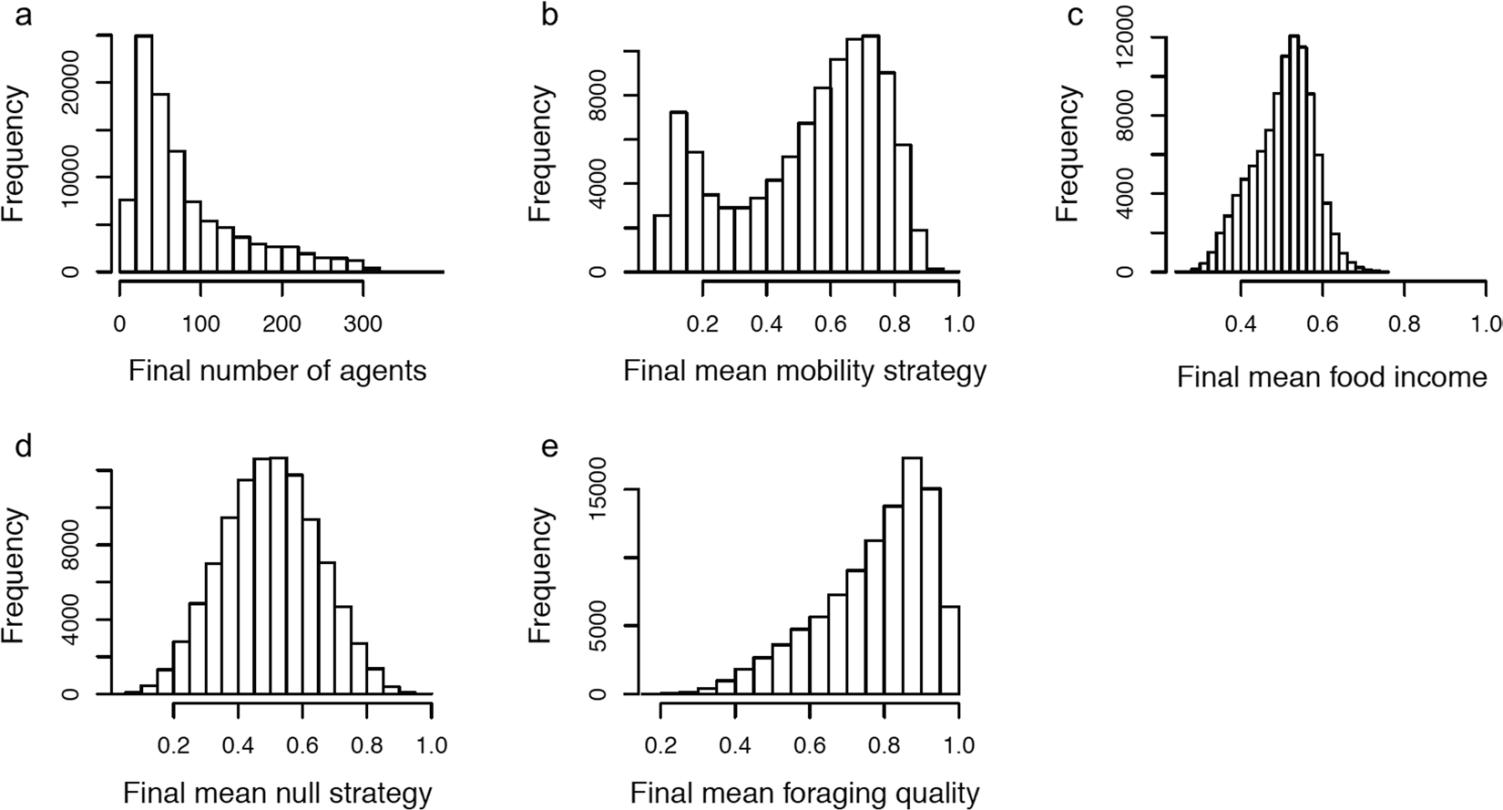
Histograms of the properties of 98,514 simulations of the model in the 1000^th^ iteration.

The distributions of the simulation outcomes (number of agents, mean strategy values, mean food income and mean foraging quality) in the final iteration are shown in Fig. 3a–e. From this we see that the distribution of final mean mobility strategies is bimodal (Fig. 3b) – some simulations have mostly mobile agents in the final iteration, and some simulations have mostly sedentary agents in the final iteration. To see how these two different types of mobility outcome co-occur with the other outcomes we develop the Outcome Clustering (OC) method described in the Methods section.

**Figure 4:**
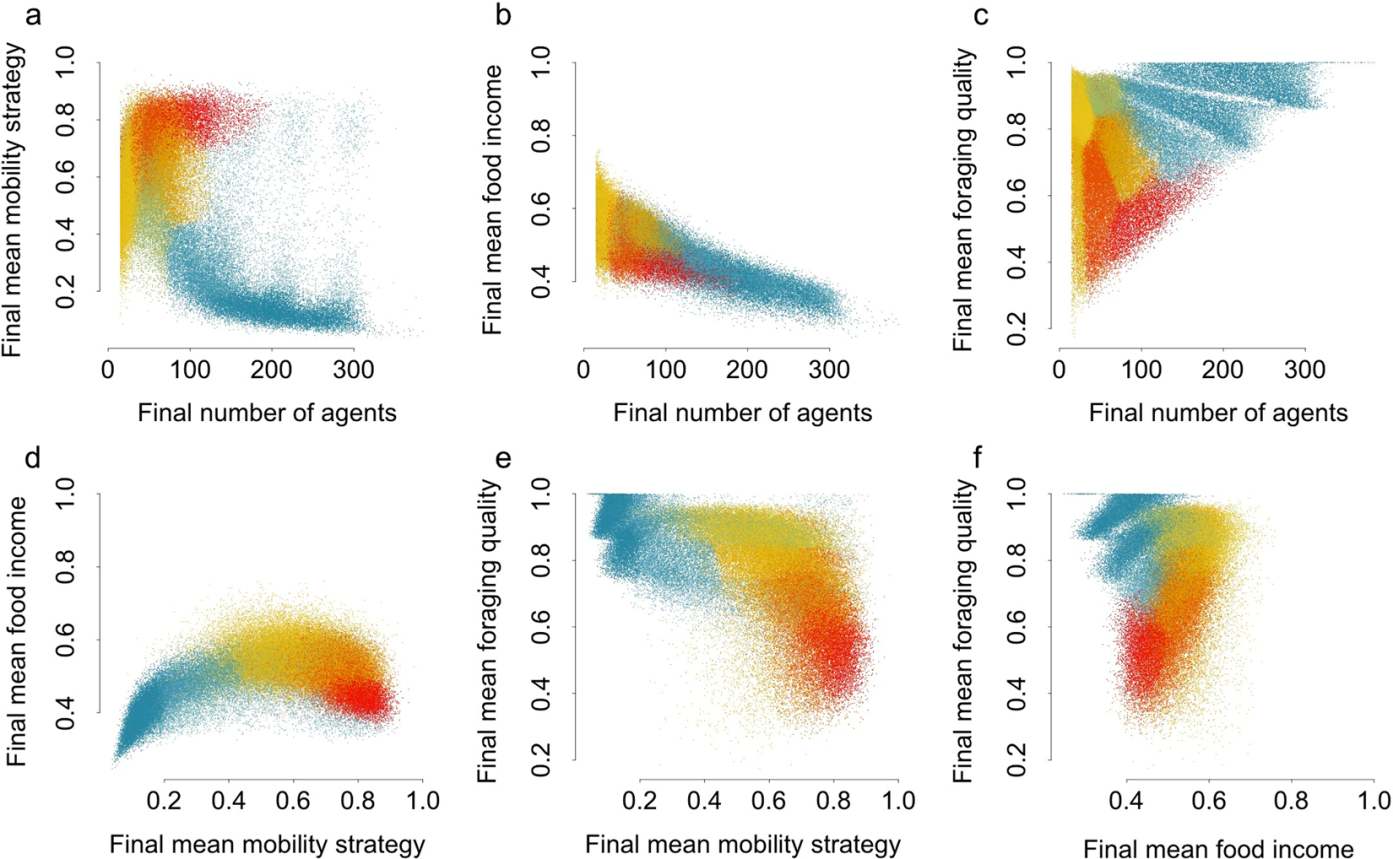
Scatter plots for each pair of outcomes. Each point represents one of the 98,514 simulations that had ≥15 agents in the 1000^th^ iteration of the model, and are coloured by the cluster the simulation was assigned to. Red represents the cluster with the most mobile agents and blue represents those with the least mobile agents.

**Figure 5:**
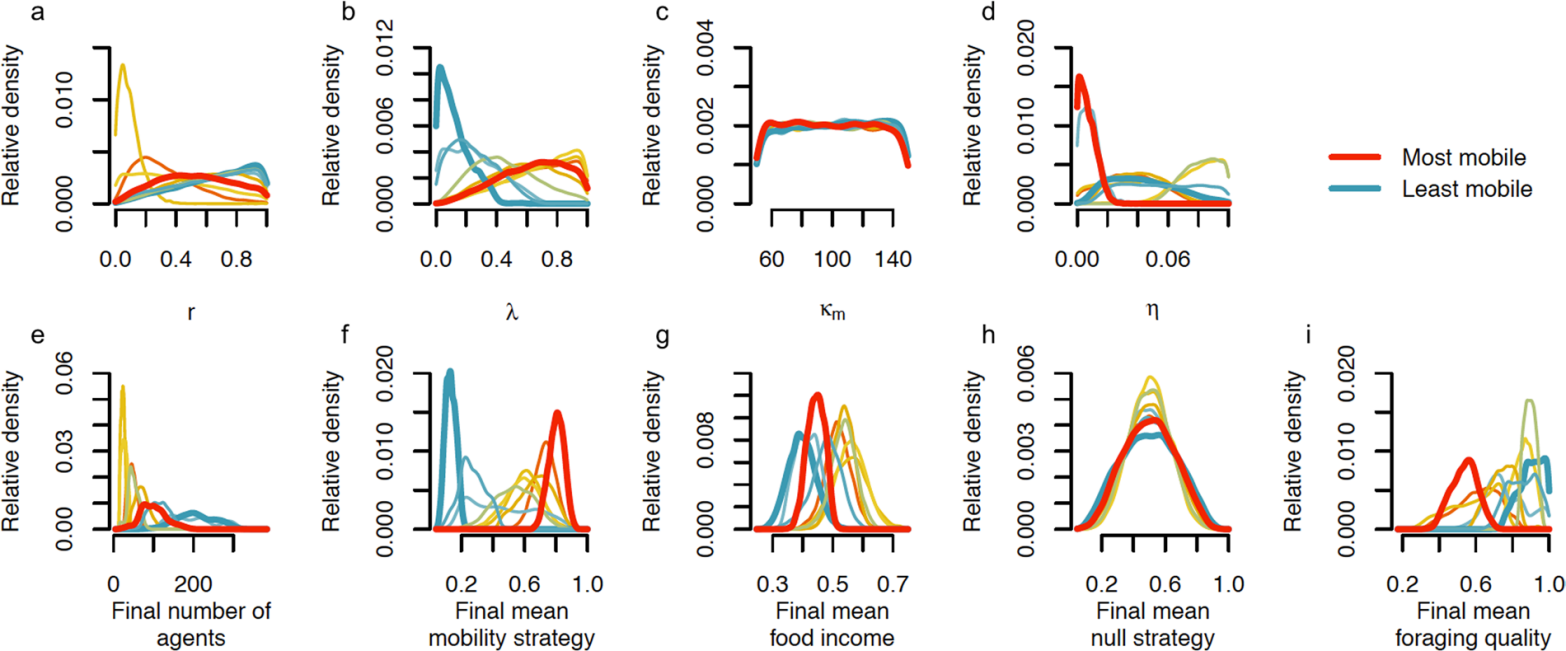
The distributions of parameters (**a**–**d**) and outcomes (**e**–**i**) for each cluster using all 98,514 simulations that had ≥15 agents in the 1000^th^ iteration of the model. The clusters with the highest and lowest mean value for the final mean mobility strategy (the red and clue lines respectively) are highlighted with a thicker line.

The nine clusters of simulation outcomes found using the OC method all had over 5000 simulations assigned to each. Table 3 gives a summary of the properties of each cluster. In Fig. 4 each pair of outcome values are plotted for each simulation and coloured by the cluster the simulation was assigned to. Figure 5 shows the distributions of values for both the parameters (Fig. 5a–d) and the outcomes (Fig. 5e–i) in each of the 9 clusters.

**Table 3:** Properties of each cluster found using the Outcomes Clustering method – the number of simulations assigned to this cluster and the mean value and 95% confidence interval for each parameter and outcome.

Cluster number	Number of simulations	Mean *r*	Mean *λ*	Mean *κ*_*m*_	Mean *η*	Mean final number of agents	Mean final mean mobility strategy	Mean final mean food income	Mean final mean foraging quality
1	6082	0.53 ± 0.006	0.64 ± 0.006	99 ± 0.7	0.01 ± 0.0001	100 ± 0.8	0.80 ± 0.001	0.44 ± 0.001	0.54 ± 0.002
2	14061	0.32 ± 0.003	0.65 ± 0.004	99 ± 0.5	0.04 ± 0.0003	47 ± 0.2	0.73 ± 0.001	0.52 ± 0.001	0.62 ± 0.002
3	15253	0.61 ± 0.004	0.59 ± 0.004	100 ± 0.5	0.04 ± 0.0003	72 ± 0.3	0.67 ± 0.002	0.54 ± 0.001	0.74 ± 0.001
4	5655	0.10 ± 0.002	0.66 ± 0.006	100 ± 0.8	0.04 ± 0.0006	23 ± 0.1	0.60 ± 0.003	0.56 ± 0.002	0.59 ± 0.004
5	18317	0.39 ± 0.004	0.70 ± 0.003	100 ± 0.4	0.08 ± 0.0002	27 ± 0.1	0.58 ± 0.002	0.56 ± 0.001	0.86 ± 0.001
6	10972	0.65 ± 0.004	0.45 ± 0.004	100 ± 0.5	0.08 ± 0.0002	50 ± 0.3	0.53 ± 0.002	0.54 ± 0.001	0.90 ± 0.001
7	5219	0.61 ± 0.007	0.26 ± 0.005	101 ± 0.8	0.01 ± 0.0002	195 ± 1.7	0.46 ± 0.005	0.42 ± 0.001	0.77 ± 0.003
8	9884	0.63 ± 0.005	0.25 ± 0.003	100 ± 0.6	0.06 ± 0.0005	115 ± 0.5	0.27 ± 0.002	0.48 ± 0.001	0.85 ± 0.002
9	13071	0.66 ± 0.004	0.13 ± 0.002	101 ± 0.5	0.04 ± 0.0004	206 ± 0.8	0.13 ± 0.001	0.40 ± 0.001	0.90 ± 0.001

The final mean null strategy does not correlate with any of the other outcomes of the model (see Table 2), whereas the final mean mobility strategy does. In Fig. 3d we see that the final mean null strategy values are always around 0.5 for all simulations, consistent with its values only being shaped by its initial random assignment and random drift, and it not influencing other model outcomes. These results reassuringly indicate that mobility strategies are evolving adaptively, rather than by random cultural drift.

The mobility strategy conservatism parameter, ${\kappa }_{m}$, makes no difference to which cluster a simulation is assigned to (Fig. 5c), and there were no correlations between ${\kappa }_{m}$ and any of the model outcomes (Table 2). This implies that our simulations are of sufficient duration for mobility strategies to evolve by selection towards evolutionarily stable strategies, and are not limited by ${\kappa }_{m}$.

Other than ${\kappa }_{m}$, the distributions of parameter values are different in each cluster (Fig. 5a,b,d). For the cluster with the least mobile agents, the foraging quality conditions are optimal; the replenishment rates of foraging quality, *r*, are high (Fig. 5a) and the depletion rates, *λ*, are low (Fig. 5b). On the other hand, for the cluster with the most mobile agents, the foraging quality conditions are poor; the replenishment rates of the foraging quality, *r*, are at a mid-range values and the depletion rates, *λ*, are high. Thus, in our model we found that low mobility evolves in good foraging quality conditions. This is consistent with Kelly’s^3^ ethnographic data; in regions of low primary biomass and high new primary productivity (i.e. where there is more to eat) there are fewer residential moves. It is also similar to one of the findings from the Hamilton *et al*.^4^ model; changes in subsistence ecology (specifically increases in biodiversity) cause switches to sedentism.

Even in low mobility clusters a high movement cost would lead to lower agent food income levels and therefore more agent death, and indeed we see that when the food income cost of movement, *η*, is large, there are fewer agents (Fig. 5d,e). In the clusters where *η* is small there are more agents, and when *η* is very low, high agent mobility strategies become more viable since there are few costs to movement.

We find that the final mean mobility strategy is negatively correlated with the final mean foraging quality and positively correlated with the final mean food income; more mobile agents generally have a higher food income. These outcomes were not predicted since there is no explicit relationship between mobility and other aspects of the model (i.e. there are no equations directly influencing an agent’s mobility strategy, and it is free to evolve), and hence the effects are indirect and multi-factored. Since there is a food income cost to movement, one may expect mobility to be negatively correlated with food income. Furthermore, since staying at one site for a number of iterations incurs a depletion cost to the site, it may also be expected that mobility would be positively correlated with foraging quality. Hence, there are some indirect mechanisms that influence the evolved mean mobility strategy. Since the density of agents is highly correlated with these outcomes, it is likely that this plays a part in these superficially counter-intuitive outcomes. As the region is bounded, inner sites will be more densely populated, and thus we expect there will be some spatial variation in the mobility strategies and food income of agents. However, in this analysis we investigate and compare the mean behaviour of the agents in different conditions and always keep the region size the same (the effect of varying this may cause more pronounced edge effects in smaller regions).

We found that some simulations give rise to agents with high mobility strategies and some give rise to agents with low mobility strategies (Fig. 3b). By using the Outcomes Clustering method we found that the simulations are not only categorizable by how high and low the mobility strategies are alone, but also by the other outcomes (Figs 4 and 5). We found that the cluster of simulations with the least mobile agents also had the highest density of agents, the lowest final mean food incomes and the highest final mean foraging quality. The cluster of simulations with the most mobile agents had the lowest final mean foraging qualities and high mean food incomes.

The outcomes of this model replicate to some extent the exponentially decreasing relationship between mobility and population density seen in the ethnographic data^3^ (see *SI Appendix*, Fig. S1). Previous explanations for this relationship include that sedentary life allowed for shorter birth intervals, as reduced mobility allows for increased energy for reproduction^19^, and that in more mobile societies more than one young child would be difficult to carry^34^. However, we find that this relationship arises without the need to invoke such processes.

The relationship between low mobility and low food income levels is interesting since the environmental quality is better in simulations generating these outcomes, so one may expect the food income levels to be higher. However, these conditions also mean that more agents can be supported, and hence the low food income reflects the fact that agent density is at or near carrying capacity (i.e. where there are a maximum number of agents at the lowest possible food incomes; also see^35–39^). While a relationship between sedentism, higher population density and poorer health (represented in part by the food income term in our model) has been observed in the archaeological record – with the onset of farming – this is usually explained by factors such as increased infectious disease loads and shifts to poorer quality diets^17–23^. Such factors may be important, but as our model suggests, they are not necessary to explain this often cited relationship.

To better understand why reduced mobility and higher population density predict lower food income – and so poorer health – in our model, we examined temporal oscillations in agent food income levels under two sets of conditions; one set that generated (a) high mobility, low population density, high food income, and another set that generated (b) low mobility, high population density, low food income. These two sets of conditions were chosen using the mean parameter values in the most and least mobile simulation clusters found from the Outcomes Clustering method (given in Table 3). We found that under conditions generating (a), food income oscillations were of greater amplitude than under conditions generating (b); the mean interquartile range of the food income values for each agent in the low mobility simulations was 0.14 and in the high mobility simulations was 0.20 (see *SI Appendix*, section 7, which gives the full details of these simulations and statistical tests). This can be explained by mobility costs; high mobility generates additional food income drain variability. Given that agents with food income <$\,{f}_{min}$ die – and death is an absorbing state – it follows that conditions generating high food income oscillation amplitudes would require higher mean food incomes than those generating low food income oscillation amplitudes in order to have an equivalent death rate. Such an explanation can be translated to real-world scenarios: To buffer against the costs and risks of being mobile, foragers require higher short-term food procurement (high food income), while in sedentary populations low short-term food procurement (low food income) is less risky as long as that food procurement is predictable (e.g. farmed food).

Thus, we can make a distinction between the traditional proximal cause explanations of reduced fitness in low mobility, high density populations (i.e. increased infectious disease loads and shifts to poorer quality diets; see 17–23), and the explanation we propose (i.e. reduced mobility costs generating reduced food income oscillations, and so lower mean food income levels required for survival). Both have the same ultimate causes^40^ in this context – the confounded processes of reduced mobility and increased population density – but the traditional explanations of reduced fitness require additional factors, ones which are not necessary in our model to generate reduced food income (which is associated with survival in our model).

## Conclusion

In this study we have added new methodological developments to the existing literature – developing a new evolutionary model of mobility decisions in hunter-gatherers with environmental interactions, including a null strategy as a means to check whether a simulated evolutionary pathway is via adaptation or cultural drift (seldom included in behavioural ecology modelling^41^, being one exception), and analysing the effects of multiple interacting parameters on a model’s behaviour using Outcomes Clustering.

Our model predicts that the evolution of sedentism in hunter-gatherers would co-occur with an increase in population density and a decrease in food income (which is associated in our model with health and survival), and it would occur in locations where the resource depletion rate is low and (to a lesser degree) the resource replenishment rate is high. Hunter-gatherers are more likely to increase mobility in places where depletion of the environment is fast and the cost of movement is low, and this would co-occur with reduced population density.

Our model predictions echo those of Bettinger’s^5^ ‘traveler-processor model’, in which, as population densities increase, hunter-gatherers shift from high to low mobility strategies that involve lower movement costs at the expense of a wider diet breadth linked to increased processing costs. Such costs are characteristic of the low-depletion high-replenishment resources – plants and small game – associated with increased sedentism in our model. If such resources are absent then mobile strategies are the only option and if the resources are over-exploited the result is likely to be periodic regional abandonment. If they are available then the decline in fast-depletion slow-replenishment resources leads to the increased diet breadth that opens the way to higher population densities based on more sedentary strategies, because the resources on which they are based support higher carrying capacities. Agriculture based on domesticated resources is at the extreme end of this continuum. Our model simulations show how these processes occur, leading to a bimodal traveller-processor outcome. In a world where some resource patches do not have the capacity to generate sustainable resources but others do, both strategies may exist side-by-side for long periods^42^.

We found that the degree of mobility conservatism makes little difference for changes in agent mobility, consistent with our simulations being of sufficient duration to allow mobility strategies to evolve by selection towards evolutionarily stable strategies, and with cultural selection on mobility being an important force. This model has replicated the known relationships between reduced mobility and high population density, and between higher environmental quality and reduced mobility, seen in hunter-gatherer groups^3^. More importantly, our model predicts poorer food incomes, and consequently health status, in more sedentary and so higher density populations, without the need to invoke additional factors such as reduced diet quality and increased infectious disease loads^17–23^. Furthermore, these results were emergent rather than being specified in the model. Given the importance of a low depletion rate for the high-density sedentary adaptation, a provocative implication of our model is that crop cultivation was simply a new means of reducing the depletion rate and increasing the replenishment rate, and that its association with reduced health status is a consequence of a more stable and predictable food procurement-consumption balance. In future studies we will show how our model can be developed to include other aspects of hunter-gatherer behaviour and environmental conditions (for example the effect of climate instability or geographical variability) and include different subsistence strategies so that predictions about the coevolution of sedentism and agriculture can be made.

## Methods

In our model ${q}_{f}\in [{q}_{f,min},1]$, where ${q}_{f,min} > 0$. The foraging quality is not allowed to reach 0 since if it did it could never grow back (see Equation 4.1).

Since there are multiple agents and sites in every iteration of the model, we need a way to summarize the properties of the agents and sites in order to understand the behavior of the overall system. We use the mean value for this. The mean outcome properties for agents and sites change over the iterations for 100 independent runs of the model, this is shown in *SI Appendix*, Fig. S5. When we further summarize each independent run of the model to investigate the effects of parameters on the outcomes we only consider the mean value in the final iteration of the model.

### Preliminary Checks

Our main interest is to investigate the effect of the parameters (given in Table 1) on the outcomes of the model after a number of iterations. However, the model outcomes may also be dependent on the values we have chosen for the constants of the model. For the region size and the number of iterations in the model, there is a trade-off between making sure that the values selected are large enough to give accurate and reproducible results, but not so large that computational costs are prohibitive.

The model was run several times to investigate the effect of initial model conditions: the number of iterations the model is run for, the region size, and the minimum foraging quality. By making changes to each of these four constraints in turn and monitoring the differences they make to the final result, we found that the overall results were still quite similar. Thus, we are confident that we are not biasing our interpretation of the results by picking random initial conditions for every run of the model, by using a region size of 10 × 10, by setting ${q}_{f,min}=0.1$, or by running the model for 1000 iterations. The analysis also shows that caution must be applied when interpreting final mean mobility strategies of around 0.5, as rather than this meaning that most agents have mobility strategies of around 0.5, it is likely to mean their strategies are distributed widely in the range of 0 to 1.

### Set up and parameter fixing

To explore how different parameters affect the model we ran it 100,000 times with randomly chosen parameter values for the foraging quality replenishment rate, *r*, the foraging quality depletion scalar, *λ*, the mobility strategy conservatism, ${\kappa }_{m}$, and the food income cost of movement per site, *η*, and recorded outcomes of the simulations after they had run for 1000 iterations. We fixed the parameters that we had data-supported values for — the maximum probability of fission, ${p}_{max}$, the maximum number of agents, ${n}_{max}$, and the probability of mutation, *μ* — and we set the initial population density to be 10% of the maximum possible (i.e. ${\rho }_{init}=0.1$).

The simulation outcomes we record are the final number of agents, the final mean mobility and null strategies of agents, the final mean food income of agents, and the final mean foraging quality of the sites. We only consider simulations in the analysis that have ≥15 agents alive in the final iteration, since otherwise taking the mean of such a small sample will generate too much noise. Thus, unless otherwise stated these are the subset of simulations analyzed here.

### Outcome Clustering Method

With the data on parameter values and the five outcomes for many independent simulations, we can investigate how parameter values influence certain model outcomes. This can be done using the Fitting to Idealized Outcomes (FIO) method^25^; for example by selecting the simulations with the highest and lowest evolved mobilities and investigating the conditions that gave rise to these states. However, there are correlation structures among multiple outcomes, and while pairwise correlation coefficients can be found (see Table 2), such coefficients will not identify non-linearity or granularity among many outcomes. For these reasons, we propose the Outcome Clustering (OC) method. This allows us to find salient groupings of outcomes, and identify the parameter combinations that shape them, permitting a more thorough exploration of model complexity without being biased by considering a single outcome.

For the OC method many simulations of the model are run, and outcomes are recorded. We then use the function ‘Mclust’ in the R library ‘mclust’^43–45^ to find clusters of simulations on the basis of four of their outcomes – final number of agents, final mean mobility strategy, final mean food income and final mean foraging quality. After identifying these simulation clusters the distributions of the parameter values in each cluster can be found. Due to processing taking a long time, we find outcome clusters based only on the first 10,000 simulations and then use these to predict the rest of the cluster classifications using the function ‘predict.Mclust’, also in the ‘mclust’ library.

## Supplementary information

Supplementary Information for Food Income and the Evolution of Forager Mobility

## Data Availability

The datasets generated during the current study are available from Figshare (https://figshare.com/articles/The_fast_and_the_fit_paper_data/7609763), and code for the model and analysis can be found on Github (https://github.com/lizgzil/agriculture-origins).
