# Supplementary material for: Food Income and the Evolution of Forager Mobility
Source: Sci Rep. 2019 Apr 1;9:5438. doi: 10.1038/s41598-019-42006-2 (PMC6443647; doi:10.1038/s41598-019-42006-2)
Supplement: Supplementary file 1 — Supplementary Information for Food Income and the Evolution of Forager Mobility [file 41598_2019_42006_MOESM1_ESM.pdf]

Food Income and the Evolution of Forager Mobility

Elizabeth Gallagher, Stephen Shennan and Mark G Thomas

Table of Contents

S1. Ethnographic data 2

S2. Assumptions of the relationship between food income and the probability of fissioning 4

S3. Modeling mutation 6

S4. Calculating site attractiveness for movement 7

S5. Site size and the maximum number of agents 8

S6. Model properties over time 9

S7. Food income oscillations 10

References 12

S1. Ethnographic data

A large dataset of information about hunter-gatherers from all over the world is provided by Kelly (1). This data includes details of hunter-gatherer mobility behavior and the environments in which the groups live. A summary of how often people move, how far they move, how dense their groups are, their mortality, and fertility rates, from the Kelly (3) dataset is shown in Table S1.

Table S1. Summaries of the cleaned data collected by Kelly (1) on the mobility, population density (Table 7-3, pg. 178–184), mortality (Table 7-7, pg. 201) and fertility (Table 7-5, pg. 195–196) of hunter-gatherer groups. Mortality here is the percentage of people under 15 years who die, and total fertility rate is the number of children that would be born per woman if they survive through childbearing years.

	Number of Groups	Mean	Minimum	Maximum	Standard Deviation
Residential Moves Per Year	52	16.5	0	75	18.7
Total Distance (km)	43	262.6	7	1600	283
Population Density (persons/100km^2)	70	34.2	0.4	266.5	47.1
Mortality (%) <15yr	27	35.3	6	61	15
Total Fertility Rate	52	5.7	2.6	8.5	1.4

Kelly's dataset shows that the number of residential moves per year exponentially decays with increasing population density – this can be seen in Fig. S1.

In a study by Mace (2) the relationship between wealth and fertility in the Gabbra nomadic pastoralists was investigated. The number of camels is used as a proxy for wealth and 848 households are included in the study. Mace found that the equations for the best-fit regression curves are

$$-0.714 + 0.516\log(Number\ of\ camels + 1)$$

and

$$-0.292 + 0.198\log(Number\ of\ camels + 1)$$

respectively for men and women's residual fertility. This result is reproduced in Fig. S2.

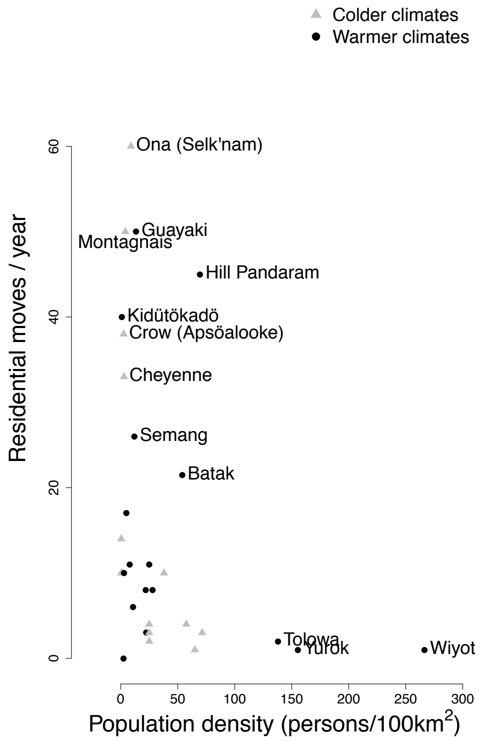

Fig. S1 Number of residential moves per year against the population density (persons per 100 km^2) for 31 forager groups. Point style denotes whether the groups live in relatively colder or warmer climates (colder climates are from the area categories `Arctic', `Subarctic/cold forests', `Temperate forests', `North American Northwest Coast', with the other area categories defined as warmer climates). The data used to make this plot is from both (1) and private correspondence with Robert Kelly.

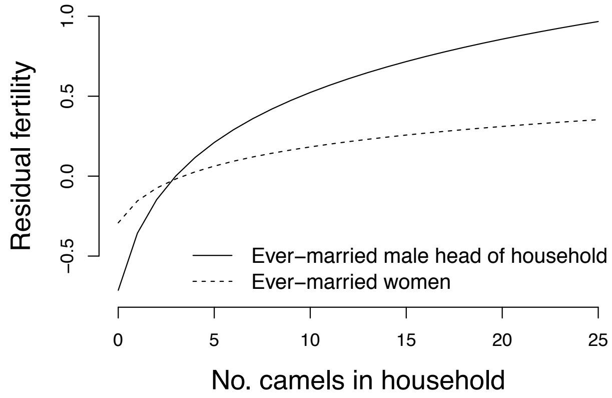

Fig. S2 The best fit regression curves of data on the number of camels in the household (a measure of wealth) and the residual fertility of ever-married male head

of household (solid line) and ever-married females (dashed line), from a study by Mace (2) on 848 Gabbra households. Reproduced from (2).

S2. Assumptions of the relationship between food income and the probability of fissioning

The model assumes new generations replace the older generations, and incest is avoided by mixing amongst different families (for example the young female members of two families swapping). We have chosen not to model these or other population dynamics (for example birth and death of individual people within the family) since these are individual dynamics and add an unnecessary level of detail.

Based on the work by Mace (2), discussed in *SI Appendix* section 1, the relationship between wealth and fertility can be modelled as

$$fertility = c + d\ log(wealth)$$

where the c and d are constants. We use this relationship between agent food income (which can be thought of as a measure of wealth) and fission probability (which should be proportional to fertility). Hence we take,

$$p = c + d\log(f).$$

The upper limit for the probability of fission, p_{max}, will be reached when agent food income is 1. Hence $p_{max} = c + d\ log(1)$, and thus $c = p_{max}$. Since the probability of fission is 0 when food income is at its minimum, f_{min}, then we can find d,

$$0 = p_{max} + d\log(f_{min}) \Rightarrow d = \frac{-p_{max}}{\log(f_{min})}.$$

Thus by substituting these we have

$$p = p_{max} - \frac{p_{max}}{\log(f_{min})}\log(f)$$

where $p_{max} \in [0,1]$.

The maximum number of agents that can survive at a site is

$$n_{max} = \max(n) = \max\left(\frac{q_{f,t-1}}{f}\right) = \frac{\max(q_{f,t-1})}{\min(f)} = \frac{1}{f_{min}}$$

and therefore $f_{min} = \frac{1}{n_{max}}$.

We can find an estimated value for p_{max} by using data on hunter-gather fertility and mortality from Kelly (1). Using this data we found that the average fertility rate of the sample of hunter-gatherer groups is 5.7, with a minimum of 2.6 and a maximum of 8.5, and the average mortality for children < 15 years old is 35.3%.

Assuming that children under 15 are not reproductive, then using the average childhood mortality, the proportion of children who survive to reproductive age is

$$1 - \frac{35.3}{100} = 0.647.$$

And thus the number of children a woman will have that survive to a reproductive age is the proportion that survives to reproductive age multiplied by the total fertility rate. From the ethnographic data this is a minimum of $0.647 \times 2.6 = 1.68$ children and a maximum of $0.647 \times 8.5 = 5.5$ children (using the minimum and maximum total fertility rates respectively).

For the family units in my model (the agents), we assume that two children need to stay in the family to replace the previous generation, but any other children can form new families. Therefore we find the family fission probability every year should be

$$\frac{Number\ of\ children\ that\ survive\ to\ a\ reproductive\ age - 2}{Generation\ time}.$$

If we set the generation time to 25, then the lower fission probability is $\frac{1.68-2}{25} = -0.0128$ (in effect this is 0), and the upper fission probability, p_{max}, is $\frac{5.5-2}{25} = 0.14$.

The modelled relationship between food income and fission probability using $p_{max} = 0.14$ can be seen in Fig. S3. The number of years until there is a new family based on these fission probabilities is simply $1/p$.

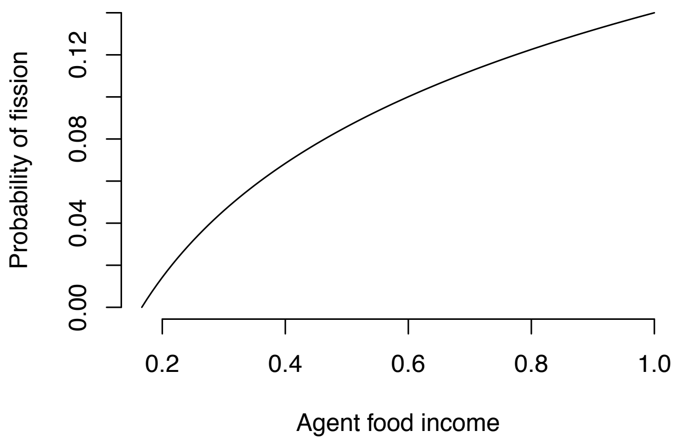

Fig. S3: Our modelled relationship between food income and probability of fission using $f_{min} = {}^1/_6$ and $p_{max} = 0.14$ based on (2) and ethnographic data in (1).

S3. Modeling mutation

When strategy mutation happens the distribution the new strategy is picked from is dependent on both the family's food income and its previous strategy.

We use a Binomial distribution to pick the new mutated strategy value from, where the distribution is influenced by the family's original strategy value, m_0, and food income, f:

$$B(n^*, p^*) = B(100 \times f, m_0).$$

This distribution means that if f is high then the distribution is narrow and if f is low then the distribution is wider and therefore the new strategy may be quite different from the original strategy. Values chosen from this distribution then need to be divided by $100 \times f$ so that the family's new mobility strategy is between 0 and 1.

Using a Binomial distribution also makes sense as a model for cultural transmission, since the value for the number of components in the distribution can be thought of as the number of components that make up mobility. However, there is the problem that when the family's strategy is 0 or 1 then the variance is 0 (variance $= n^* p^* (1 - p^*)$).

Thus there could be fixation to only either mobility strategies of 0 or 1 if these values ever occur. Hence, to avoid this issue we pick the strategies from between [0.01,0.09]. If a strategy value of < 0.01 or > 0.99 is selected then it is set to 0.01 or 0.99 respectively.

S4. Calculating site attractiveness for movement

If a family moves site the site it moves to is determined by proximity and site quality. All the site 'attractiveness' scores, A, are calculated and the site the family moves to is then picked weighted by these values. The attractiveness of site b to a family that was previously at site a is calculated as

$$A_b = f_b^*(1 - \frac{d_{a,b}}{d_{max}})$$

where $d_{a,b}$ is the distance between site a and site b, and d_{max} is the maximum distance possible in this region. f_b^* is the potential food income a family could have at site b and is calculated as

$$f_b^* = \frac{q_f^b}{n_b + 1}$$

where q_f^b is the foraging quality of site b and n_b is the number of agents at site b.

A visualization of calculating site attractiveness can be seen in Fig. S4.

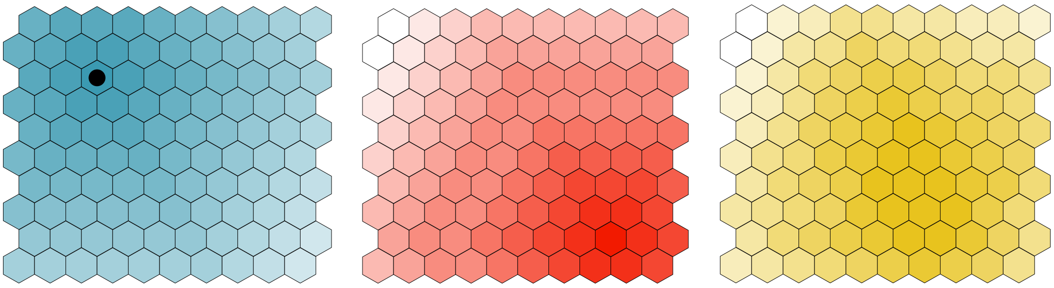

Fig. S4: An example of the distances (left) from the original site (black circle), the potential food income (middle) and the attractiveness score calculated from these (right). Bolder colours represent the distance being closer (blue), or a higher food income (red) or attractiveness (yellow).

S5. Site size and the maximum number of agents

We can estimate the size of the region we are modelling from the ethnographic data found in (1). We found that the maximum total distance moved each year by the hunter-gatherer groups in the dataset given in (1) is 1600 km. Therefore in this model we want the maximum possible distance moved in an iteration of the model to be less than or equal to 1600 km. In a region of 10×10 hexagons the maximum number of moves possible is 15.

Using basic trigonometry the distance from the centre of one hexagon to the centre of a neighbouring hexagon is $\sqrt{3}a$ where a is the length of a side of the hexagon.

Therefore in this model the following inequality should hold

$$(15-1)\sqrt{3}a \leq 1600km.$$

The maximum population density of the hunter-gatherer data in (1) is 2.665 persons/km^2 and the minimum is 0.004 persons/km^2. Thus we want the model to have a similar range in densities.

The maximum population density will occur when there are n_{max} agents at every site and the minimum will occur when there is one agent in the entire region. I will assume a family has four members, and therefore the number of people is 4 multiplied by the number of agents. Thus for the maximum population density

$$\frac{4n_{max}}{H} = 2.665 \text{ persons/km}^2$$

and for the minimum population density

$$\frac{4}{s_x s_y H} = 0.004 \text{ persons/km}^2,$$

where H is the area of a hexagon,

$$H = \frac{3\sqrt{3}}{2}a^2.$$

In a region of 10×10 hexagons then $s_x s_y = 100$ and by rearranging these equations $H = 10$km^2 and $n_{max} = 6.66$. Since we must have a discrete value for n_{max} and we want the population density to fall in the observed range of [0.004, 2.665] persons/km^2 then we will use $n_{max} = 6$ as a default value.

Using these values we can rearrange these equations to find $a = 1.96$km. Thus using a 10×10 region we are modelling a region of 1000 km^2 which can theoretically support a maximum of 600 agents (although practically this will be limited by other parameters).

S6. Model properties over time

The number of agents, mean mobility strategy, mean food income and mean foraging quality change every iteration of the model. Furthermore, each time a simulation of the model is run the behavior is different. The variation in different simulations and the simulation dynamics over time are shown in Fig. S6.

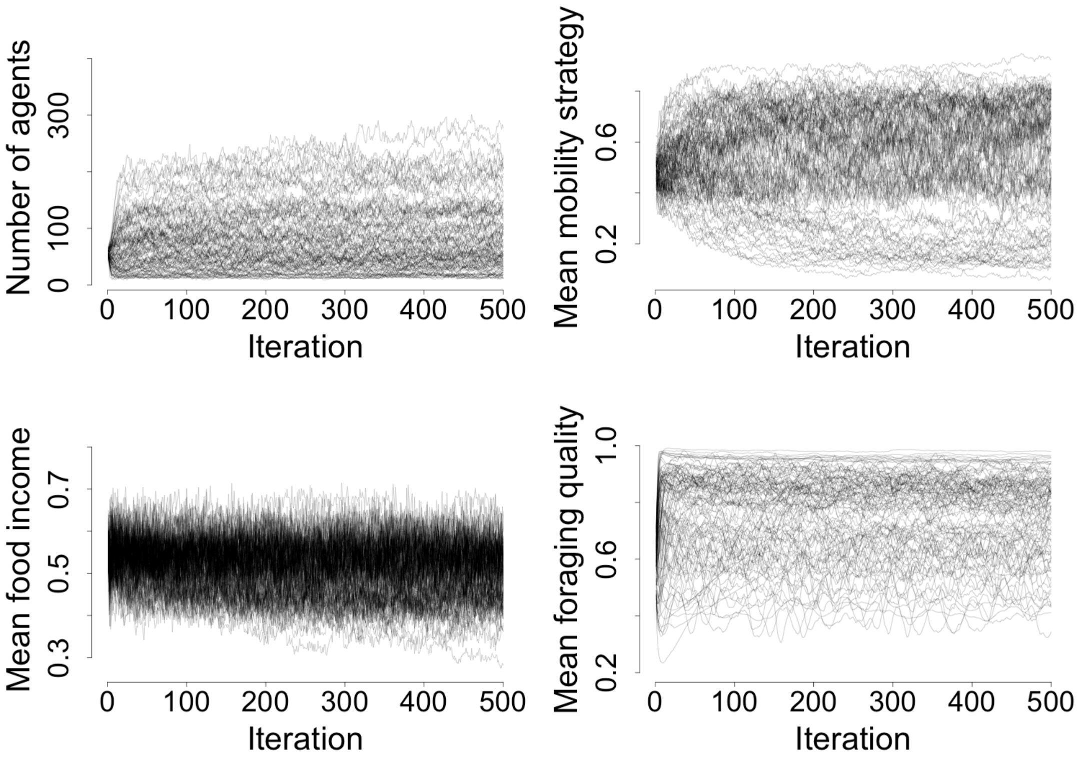

Fig. S6. The number of agents, the mean mobility strategy of all agents, the mean food income of all agents and the mean foraging quality of all sites, at each iteration of the model, run for 100 simulations of the model (each line).

S7. Food income oscillations

We ran the model 50 times with low mobility parameter values and 50 times with high mobility parameter values – shown in Table S2. With the exception of κ_m these parameter values were the mean values found in the most and least mobile simulations (Table 3, main text).

Table S2. Parameter values chosen to study food income oscillations in high and low mobility simulations.

	r	λ	κ_m	η
Low mobility simulations	0.66	0.13	100	0.04
High mobility simulations	0.53	0.64	100	0.01

For each of these simulations all the food income and mobility strategies of agents were saved at every iteration. A random sample of 100 agents from each of these simulations were chosen to look at. Since not all simulations ever had 100 agents, in total 4842 agents from the low mobility simulations and 4520 agents from the high mobility simulations ended up being investigated. How food income changes over time can be seen for each agent in Fig. S7.

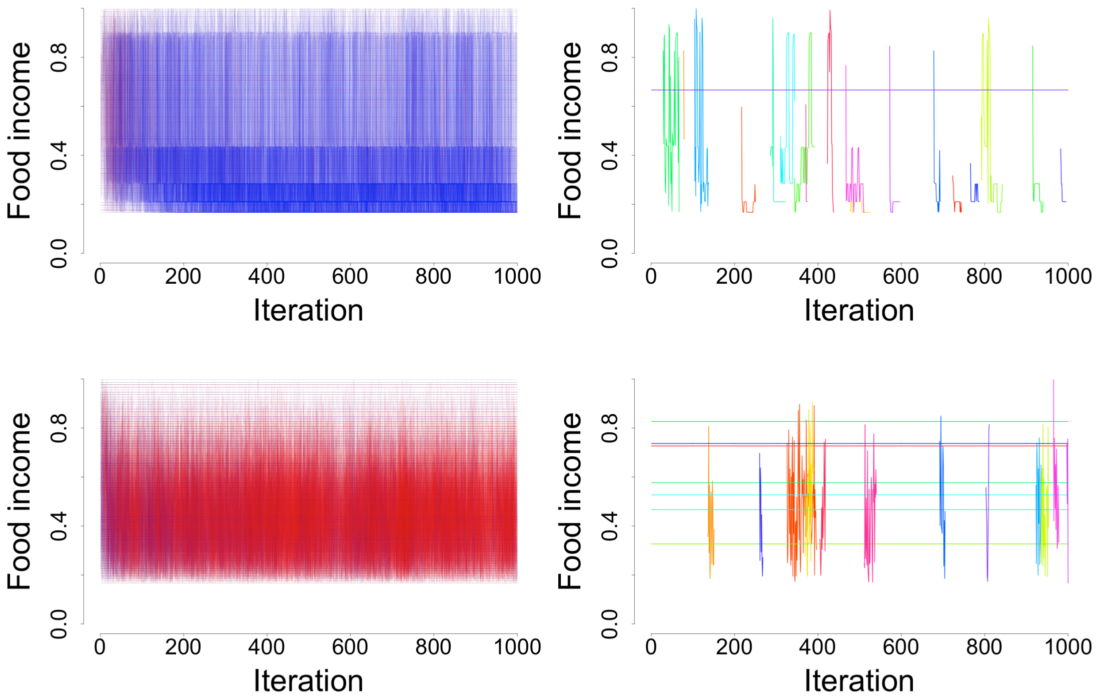

Fig. S7. Food income values over time for agents in the low mobility simulations (top row) and high mobility simulations (bottom row). Food income values for all agents in the sample are shown in the plots on the left, where colour denotes the final mobility strategy each agent had – blue being 0 and red being 1. Food income values for a random sample of 20 agents are shown in the plots on the right, where colour is used to distinguish the values of each agent.

The interquartile range (IQR) of the food income values (the difference between 75th and 25th percentiles), the mean food income value and the mean mobility strategy were calculated for each agent in each of the low and high mobility simulations. The distributions of these values can be seen in Fig. S8 and Fig. S9. From these we see that the IQR for food income is generally higher in the agents in the high mobility simulations than the agents in the low mobility simulations. The mean IQR of the food income values for each agent in the low mobility simulations is 0.14 and in the

high mobility simulations is 0.20. A Welch two sample t-test reveals there is a statistically significant difference in these means (t-score = -22.7 and p-value < 2.2e-16).

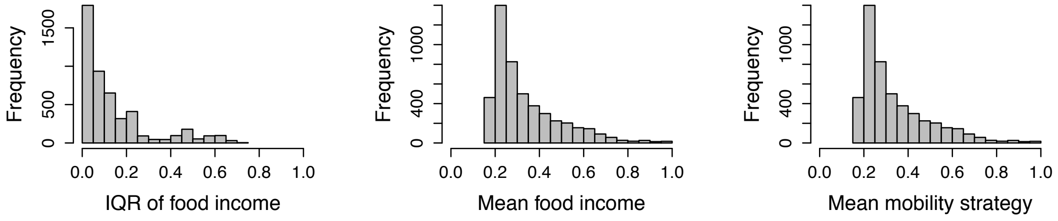

Fig. S8. Histograms of the interquartile range (IQR), mean food income and mean mobility strategies of all 4842 agents in the low mobility simulations.

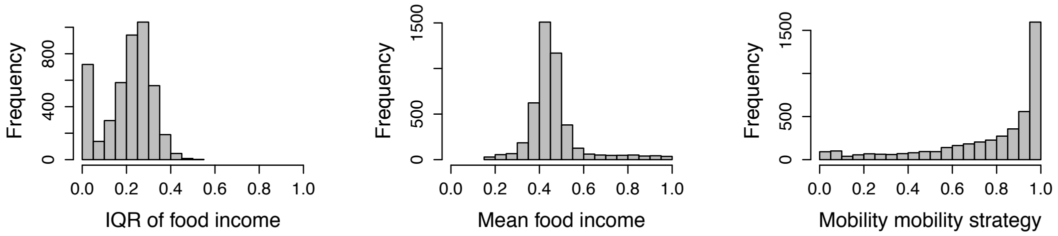

Fig. S9. Histograms of the interquartile range (IQR), mean food income and mean mobility strategies of all 4520 agents in the high mobility simulations.

References

1. Kelly RL (2013) *The lifeways of hunter-gatherers: The foraging spectrum* (Cambridge University Press, Cambridge).

2. Mace R (1996) Biased parental investment and reproductive success in Gabbra pastoralists. *Behav Ecol Sociobiol* 38(2):75–81.
